# Enhancing Beef Hamburger Quality: A Comprehensive Review of Quality Parameters, Preservatives, and Nanoencapsulation Technologies of Essential and Edible Oils

**DOI:** 10.3390/foods14020147

**Published:** 2025-01-07

**Authors:** Tainara Santos Oliveira, Rogeria Comastri de Castro Almeida, Vanessa de Lima Silva, Cláudio Vaz Di Mambro Ribeiro, Leilson Rocha Bezerra, Camila Duarte Ferreira Ribeiro

**Affiliations:** 1 Nutrition School, Federal University of Bahia, Rua Basilio da Gama s/n, Canela, Salvador 40110-907, BA, Brazil; tainara@ufba.br (T.S.O.); rogeriac@ufba.br (R.C.d.C.A.); 2 Graduate Program in Food Science, College of Pharmacy, Federal University of Bahia, Rua Barão de Jeremoabo, 147, Ondina, Salvador 40170-115, BA, Brazil; vanessa.lima@ufba.br (V.d.L.S.); claudioribeiro@ufba.br (C.V.D.M.R.); 3 Department of Animal Science, Federal University of Campina Grande, Patos 58708-110, PB, Brazil; leilson@ufpi.edu.br

**Keywords:** meat preservation, oxidation, storage, nanoparticles, antioxidants, essential oil, edible oil

## Abstract

Essential and edible oils have applications in reducing oxidative processes and inhibiting the growth of microorganisms in meats and their derivatives, providing a natural alternative to synthetic preservatives. This preservative action meets the demand for clean labels and safe products, aiming to replace synthetic additives that pose potential health risks. Advances and limitations in applying essential and edible oils in meat preservation, highlighting their preservative properties or ability to improve nutritional profiles, are explored in this study. Despite the benefits, the direct application of oils faces limitations such as low solubility and sensory impact, which can be overcome by nanotechnology, including association with biopolymeric matrices, focusing on the protection of bioactive compounds and enhancing the functionality of natural oils in food systems. This approach is essential for innovation in food preservation, promoting safety and sustainability in the meat sector, and following consumer expectations and food safety guidelines. Studies suggest that by combining the functional benefits of essential and edible oils associated with nanotechnology, there can be significant contributions to innovation and sustainability in the meat sector, promoting natural preservation and meeting market regulations and expectations.

## 1. Introduction

Meat is consumed worldwide, with global production expected to reach 60.4 million tons by 2024 [1] and is appreciated for its nutritional value, distinctive flavor, and the variety of cooking techniques that can be applied to it [2]. Its flexibility allows it to be offered in various shapes and cuts, as well as transformed into processed products like burgers. Its high nutritional value, rich flavor, and diverse preparation methods allow it to be available in various cuts and forms, including burgers, which are popular for their convenience [2]. However, the grinding processes involved in their production increase the exposed surface area, increasing the risk of microbial contamination and accelerating the oxidation of lipids, proteins, and pigments [2,3].

Microbial contamination and lipid oxidation are the primary causes of deterioration and consequent loss of nutritional quality, sensory degradation, and texture alteration in meats and meat products [4]. Such undesirable changes can be effectively controlled or minimized using natural antioxidants (essential oils, spices, flavonoids, catechins, rosemary antioxidants, and herbal extracts) as substitutes for synthetic preservatives in fresh/processed meat during refrigerated storage, a topic of interest for global public health [5].

The food industry widely employs synthetic antioxidants, such as EDTA (ethylenediaminetetraacetic acid), TBHQ (tert-butylhydroquinone), PG (propyl gallate), BHA (butylated hydroxyanisole), BHT (butylated hydroxytoluene), and sodium erythorbate, to minimize or inhibit oxidative reactions [6]. However, concerns about their toxicological effects have driven the demand for natural antioxidants, explored not only for their antioxidant properties but also as alternatives against the emergence of multi-resistant bacteria [7,8]. Additionally, consumers have opted for clean-label food products subjected to minimal processing and free from synthetic preservatives [9]. Essential and edible oils stand out as alternatives for this application purpose, mainly due to their antimicrobial and antioxidant properties.

Essential oils (EOs) are secondary metabolites of various aromatic plants, consisting of sesquiterpenes, terpenes, and diverse oxygenated compounds like phenols, aldehydes, lactones, esters, ketones, phenolic ethers, and alcohols. These oils hold significant industrial value and are widely utilized in various fields, including agriculture, cosmetics, nutrition, and pharmaceuticals. Their importance stems from a broad range of biological activities, such as antimicrobial, anti-inflammatory, antioxidant, analgesic, and repellent properties, among others. Furthermore, they are recognized as safe substances by the Food and Drug Administration (FDA) [10], which increases interest in their use as safe food additives and natural preservatives.

EOs are defined by the International Organization for Standardization as substances obtained from plant materials through methods such as dry distillation, steam distillation, or mechanical extraction of citrus fruit peels, followed by the separation of the aqueous phase, if present, using physical methods [11]. The antimicrobial and antioxidant activities are associated with the volatile compounds present in these oils, used for various applications, where they can act as bactericides, fungicides, antiparasitics, and antioxidants, among others [12,13,14]. However, the use of these oils as preservatives and antioxidant agents in the food industry still faces problems due to characteristics such as low water solubility, the possibility of aggregating sensory attributes to food when added in high concentration, the sensitivity of bioactive compounds to oxygen, light, and heat during processing, use, and storage [9]. Therefore, nanoencapsulation presents itself as a significant alternative as well as a rapidly expanding technology to protect substances at the nanoscale (<1000 nm), especially bioactive compounds [15]. Encapsulation of oils is viable and efficient and is an approach to overcome challenges faced in its application, increasing bioavailability, solubility, and chemical and thermal stability; reducing volatility; and improving the delivery system of antioxidant compounds in oils [16].

The effects of applying nanoencapsulated oils in extending the shelf life of burgers, with control over lipid oxidation and microbial growth, are predominantly described for EOs, as reported in burgers added with EO nanoparticles from cumin seed [17], lemon [18], and *Zataria multiflora boiss* [19]. However, when applied to burgers, vegetable oils are preferably investigated concerning partial fat substitution, with nutritional impacts, especially in improving the product’s lipid profile [20,21,22].

This review systematizes the knowledge regarding the application of essential and edible oils in meats and meat products, particularly burgers. Quality parameters and the preservative effect of bioactive compounds applied to meats and their derivatives, addressing the preservative properties of these oils, including the use of nanoencapsulation as a strategy to mitigate challenges related to oxidative instability, sensory properties, and low solubility of oils, are discussed.

## 2. Definition, Production, History, and Quality Parameters of Beef Burger During Refrigerated Storage

Originally considered secondary options, burgers have become a convenient and widely appreciated choice among consumers. Furthermore, they are recognized for providing high-quality protein and serving as an efficient solution for the full utilization of all parts of the animal [23].

Although there is no standard international regulation defining what constitutes a hamburger, many countries have established guidelines detailing its composition. According to the code of federal regulations (CFR), a hamburger is a meat product made from fresh and/or frozen ground beef that may or may not include the addition of beef fat and seasonings. Water, phosphates, binders, or extenders are not allowed, and the product may contain a maximum of 30% fat [24]. In Brazil, hamburgers are defined by the technical regulation of identity and quality (RTIQ), governed by ordinance SDA No. 724. This standard describes hamburgers as a product derived from minced meat obtained from slaughtered animals, with the optional inclusion of adipose tissue and other components. The product is shaped into an oval or circular form and subjected to a specific technological process [25]. Regarding physicochemical characteristics, the legislation allows for the presence of up to 3% total carbohydrates, at most 25% fat, and requires a minimum of 15% protein [25]. Furthermore, this regulation allows for the addition of vegetable oils as optional ingredients in the formulation of hamburgers.

The hamburger gained popularity in Germany during the 19th century, in the city of Hamburg, enjoyed raw, in the form of patties with various seasonings, known as “Hamburg steak”, similar to steak tartare preparations [26]. From the 1920s, it emerged in the United States, becoming an icon of the American lifestyle. Due to the exponential growth in the burger restaurant sector, it is essential to observe every stage of the production process that might risk consumer health [27]. Moreover, sensory properties play a crucial role in consumer satisfaction and purchase decisions, with attributes such as juiciness, color, texture, odor, and flavor being the most important [28].

In terms of quality, two main reasons lead to the reduction of shelf life and acceptability of burgers [9]: the oxidation of fatty acids and the presence of microorganisms responsible for food spoilage are significant concerns with this type of product. Raw burgers carry a higher risk of microbial contamination due to the grinding process, which increases the exposed surface area, and even when refrigerated, microorganisms can continue to multiply [23,29]. Additionally, failures in improper hygiene conditions, inadequate storage, unsuitable packaging, exposure to unfavorable environmental conditions, repeated freezing and thawing cycles, improper cutting, and improper transportation of raw materials can also contribute to contamination. Given that a significant number of consumers prefer burgers cooked medium-rare, this practice may pose health risks due to foodborne illnesses [30].

## 3. Factors Affecting Meat and Meat Product Quality: Microbial Growth, Oxidative Processes, Color and pH Changes

### 3.1. Microbial Growth

Due to the availability of nutrients, high moisture, and pH with an optimal range for microbial growth, fresh meat and products are highly perishable and favorable to the growth of spoilage and pathogenic microorganisms [28,31]. Spoilage bacteria can have their development controlled by adopting measures focused on reducing contamination from processing and application of growth inhibitors. In contrast, the presence of pathogenic bacteria is limited to consumption, and their control requires measures that prevent their contamination during the slaughter stage and avoid cross-contamination with contaminated carcasses [32].

The factors that affect microbial growth in meat products include competition between microorganisms, the physical properties of the meat, pH (acidity or alkalinity), redox potential, the presence or absence of oxygen, humidity, and the combination of time and temperature [33]. Additionally, despite the use of antimicrobial compounds to extend the shelf life, processes such as thermal treatment, vacuum packaging, freezing, and refrigeration may not fully ensure the product’s safety. Therefore, antimicrobial molecules are frequently employed to increase food shelf life [33].

The main spoilage bacteria associated with meat and products are *Proteus*, *Leuconostoc*, *Lactobacillus*, *Enterobacter*, *Moraxella*, *Brochothrix thermosphacta*, *Acinetobacter*, and *Pseudomonas* [31]. The presence of these spoilage bacteria causes negative changes in the meat product appearance, producing undesirable odor, texture, flavor, and color [34]. Besides microbial spoilage, meat and meat products are also prone to contamination by pathogenic microorganisms. According to Hui et al. [35], nine main pathogenic bacteria are associated with meat and meat products, such as *Salmonella* spp., *Campylobacter jejuni*, *Escherichia coli* O157:H7, *Clostridium perfringens*, *Clostridium botulinum*, *Listeria monocytogenes*, *Staphylococcus aureus*, *Bacillus cereus*, and *Yersinia enterocolitica*. Oliveira et al. [36] reported contamination by *L. monocytogenes* in raw chicken meat, predominantly in packaged chicken cuts. The International Commission on Microbiological Specifications for Foods (ICMSF) [37] establishes microbiological standards for hamburger beef as criteria for determining shelf life and meat quality permitted for human consumption as follows: the limit for mesophilic aerobes is 10^7^ colony-forming units (CFU)/g for positive coagulase *S. aureus*, it is 10^2^ CFU/g; for *Escherichia coli*, it is 10^2^ CFU/g; and it determines the absence in 25 g for *Salmonella* sp. There has been an observed increase in foodborne diseases related to microbial contamination. The World Health Organization (WHO) estimated, in the last report issued in 2015, that every year around 600 million cases (nearly 1 in every 10 people in the world) of foodborne diseases occur and 420,000 associated deaths worldwide [38]; and following Resolution 73.5 of the World Health Assembly (WHA 73.5), the WHO will need to issue a new report by 2025 on the global landscape of foodborne diseases, with updated estimates [39]. Moreover, according to the Centers for Disease Control and Prevention (CDC) [40], beef is a food whose consumption is associated with outbreaks of diseases, with recalls of ground beef in American states reported. In the USA, G1 reported in 2007 the recall made by Cargill of nearly 500,000 kg of hamburger meat due to suspected *E. coli* contamination. This microorganism is responsible for causing serious symptoms of food infection like diarrhea and vomiting and can be linked to a lack of hygiene during handling [32].

### 3.2. Lipid Oxidation

Although hamburgers are a low-cost and convenient product, they are highly susceptible to deterioration caused by oxidation or microorganism contamination. Ground meat is prone to lipid, protein, and pigment oxidation due to its increased surface area exposed to oxygen. Additionally, the grinding process causes the rupture of muscle cells, breaking their membranes and releasing prooxidant ions. This results in the interaction between oxidizing agents and unsaturated fatty acids, leading to spoilage, reduced quality, shortened shelf life, and potential health risks [41,42]. The oxidation of lipids leads to a reduction in the quality and safety of food products, impacting attributes such as color, flavor, texture, and nutritional content by depleting vitamins and essential fatty acids. Over time, this process can result in the formation of potentially mutagenic and/or carcinogenic compounds [9].

Most lipids contain fatty acids, divided into two types: saturated and unsaturated. Although unsaturated fatty acids are more beneficial for the organism, their presence in food decreases shelf life due to higher susceptibility to oxidation. The double bonds are more susceptible to reactions with reactive species, starting the oxidative process [43,44]. Lipid oxidation can occur due to enzyme hydrolytic action (e.g., lipases), heat, high moisture or water activity levels, enzymatic oxidation (e.g., through lipoxygenase action), photo-oxidation (i.e., UV radiation), and auto-oxidation (e.g., by free radicals in the presence of oxygen). Therefore, lipid oxidation is of significant economic concern in the food industry [45].

Meat and its derivatives are highly susceptible to oxidation because membrane lipoproteins release phospholipids. Phospholipids are the primary source of polyunsaturated fatty acids and are the lipid class most contributing to oxidative flavor deterioration, while triglycerides are found in all fats and oils [43].

The process of lipid oxidation involves a complex interaction where unsaturated fatty acids predominantly react with molecular oxygen via a free radical chain mechanism. This reaction unfolds in three overlapping stages: initiation, propagation, and termination, creating a continuous chain reaction. The primary auto-oxidation is followed by secondary reactions leading to lipid degradation and oxidative rancidity development. The major unsaturated fatty acids composing animal tissue lipids are oleic, linoleic, linolenic, and arachidonic acids. Their auto-oxidation gives rise to various hydroperoxides, leading to the formation of many volatile compounds [44,45].

Thus, the oxidation of unsaturated fatty acids from phospholipids and triacylglycerols restricts the shelf life and acceptability of meat [9], as it results in oxidative rancidity with the production of undesirable volatile substances like ketones, aldehydes, alcohols, acids, and hydrocarbons responsible for unpleasant taste and odor [46], besides negatively altering the color and appearance [31]. Lipid oxidation and changes in muscle pigments are critical indicators of meat quality, particularly concerning spoilage, as lipids and proteins are susceptible to damage by reactive oxygen species [28,47]. Both intrinsic and extrinsic factors influence the extent and speed of lipid oxidation. These factors include pro- and antioxidant content and activity, endogenous ferrous iron presence, myoglobin, enzymes, pH, temperature, ionic strength, oxygen consumption reaction, oxygen contact surface area, water activity, and meat fatty acid composition, which varies depending on several factors, including animal diet [48].

The determination of thiobarbituric acid reactive substances (TBARS) is used as an indicator to assess the amount of secondary lipid oxidation products closely related to meat sensory quality [9]. A malondialdehyde (MDA) value of 1.0 mg per kilogram of meat is considered a consumer rancidity perception threshold [49].

### 3.3. Protein Oxidation

Another important oxidative process in meats and their products is protein oxidation. Proteins are important diet constituents as they are a major source of essential amino acids and, in the case of meat, are primarily responsible for texture [43]. The three most important chemical modification reactions in food proteins are protein oxidation, glycation, and lipo-oxidation (reaction of lipid peroxidation products with proteins). These individual protein modification reactions occur simultaneously in foods [50]. The meat protein’s susceptibility to oxidation reactions alters its functional properties and consequently decreases food quality. The reduction in nutritional quality occurs through the loss of essential amino acids due to lysine, methionine, and tryptophan degradation, reducing the protein value of foods. The bioavailability decreases as cross-linking between oxidized proteins can reduce protein digestibility, making nutrients less accessible for body absorption [42].

The oxidative stress results in modifications in amino acid side chains and protein chains (attacking the polypeptide backbone), fragmentation, aggregation, and polymerization of the protein. These effects result in both biochemical and structural disruptions, leading to several sensory, technological, and nutritional issues in animal-origin foods [42,50]. Additionally, protein oxidation in animal-origin foods can be directly induced by reactive oxygen species (ROS) (whether free radicals or not) and reactive nitrogen species (RNSs) or indirectly by lipid oxidation secondary products [42,43]. Depending on their reactivity, ROSs react with biomolecules more or less selectively and, in protein cases, affect different molecular sites [50].

Protein oxidation progresses and concludes via various mechanisms, depending on the specific target and the type of oxidizing agent involved [42]. This process promotes carbonyl formation, the major oxidation protein result, loss of sulfhydryl groups, hydroperoxides, and sulfoxides. It also causes fragmentation due to peptide bond cleavage, protein aggregation by cysteine or tyrosine residue cross-linking, proteolytic enzyme inactivation, and decreased protein solubility; consequently, leading to less juiciness and tenderness of the meat, as maturation and tenderization depend on protein hydrolysis by proteolytic enzymes, i.e., cathepsins and calpains [43]. To perform necessary activities in the human body, proteins must be in an intact chemical and structural state. In the food industry, conformational changes suffered by oxidized proteins are more influential in modifying food color and texture [42].

The efficiency of protein digestibility can be impacted by oxidative modifications in enzymatic recognition sites on amino acids, impairing digestive enzymes’ action. This alteration depends on the ROS type initiating oxidation, the target amino acid, the resulting modifications, and the specificity of digestive enzymes. Oxidation of amino acids can also affect their bioavailability, making certain amino acids unsuitable for absorption and protein synthesis [42].

Sulfhydryl group loss is a modification type that can be used as a protein oxidation marker in meats, as amino acids like cysteine and methionine are very sensitive to almost all ROS, and their loss in meat systems can reflect specific oxidative damage to meat proteins. As a result of protein oxidation, sulfhydryl groups form inter- and intramolecular disulfide bonds, and then the concentration of sulfhydryls decreases with oxidative reactions’ progress [51]. Therefore, sulfhydryl concentration determination is an appropriate indicator for evaluating protein oxidation levels. The increase in sulfhydryl content may be associated with the greater exposure of this compound due to protein degradation by enzymatic or microbial action. Normally, sulfhydryl groups are located inside the protein due to the relatively high hydrophobicity of the surface. Upon exposure, these groups become targets for radical attack [52], and then the reduction can be attributed to direct radical attacks on thiol groups in meat protein cysteine residues, converting them into disulfide bridges and other oxidation products, such as sulfenic, sulfinic, and sulfonic acid and thiosulfate [53], as well as possible interactions with MDA, the lipid oxidation product, reducing free thiol groups during storage [52].

### 3.4. Color and pH Changes

**Figure 1:**
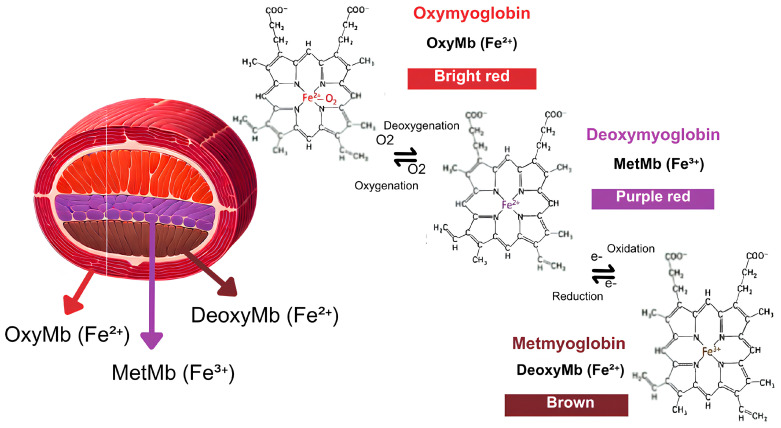
Meat colors and main form of myoglobin in muscle. Source: Adaptaded from Bak et al. [54].

In addition to lipid and protein oxidations, meat color, and appearance influence customer decisions to select or reject products [9]. Many factors determine or influence meat color such as slaughter quality, muscle metabolic profile, animal species, age, environment, types and amounts of pigments present in the muscle, types of fibers that compose the muscle and thus determine light dispersion degree and penetration depth, besides intramuscular fats and surface dehydration, which confer different gloss degrees, affecting light dispersion and reflection [35,48]. The most influential pigment in meat color is myoglobin, contributing to color depending on its redox state and concentration [48]. Myoglobin is a globular protein that stores oxygen (O_2_) in the muscle. It is made up of a single polypeptide chain known as globin (apoprotein) and a prosthetic heme group for O_2_ binding, containing an iron atom at its core [35]. This heme group gives myoglobin and its derivatives distinct colors based on chemical structure changes in the iron atom [48]. There are three main forms of myoglobin in muscles (Figure 1): deoxymyoglobin (Mb2+), oxymyoglobin (OMb2+), and metmyoglobin (MMb3+), each conferring different colors on meat [35].

In the presence of O_2_, the iron of deoxymyoglobin remains in the +2 (Fe^2+^) ferric state, which is the lowest oxidation state, conferring a dark purple-red color to the tissue [35]. The oxymyoglobin oxygenation of deoxymyoglobin can be observed when some parts of the meat, appearing dark, are exposed to the atmosphere after package film removal, causing a temporary reduction in local O_2_ concentration and presenting a bright red hue [35]. The oxidation of oxymyoglobin (Fe^2+^) to metmyoglobin (Fe^3+^) is responsible for the discoloration of meat during storage. This determines a color change in the meat to brown, and its extension is related to the proportion of metmyoglobin formed. Meat browning produces a rejection reaction in the consumer. The main mechanism for myoglobin oxidation is associated with the occurrence known as the Fenton reaction, where ferrous iron (Fe^2+^) can react with molecular oxygen to produce superoxide anion (O_2_•^−^), with concomitant oxidation to ferric iron (Fe^3+^) [35,48]. Metmyoglobin formation is often characterized by an auto-oxidation process that can be intensified by acidic pH, temperature, increased muscle post-mortem free radicals, microbial growth, or during meat processing [35].

Packaging influences meat color due to different gas permeabilities, moisture in the packaging film, and the type of gas mixture introduced in modified atmosphere packaging cases. The function of various packaging types is not only to extend product shelf life but also to maintain a good appearance, control transpiration, and preserve or enhance the most desirable meat color shades. In vacuum packaging, fresh meat acquires a dark and purplish color due to O_2_ deprivation. However, once the meat is unpacked and re-exposed to the atmosphere, its color converts to red in a relatively short time due to rapid oxymyoglobin formation [35].

Besides the various myoglobin forms’ ability to catalyze lipid oxidation in an acidic environment, HUI et al. [35] highlighted that transition elements, among them iron, can catalyze the decomposition of peroxides, generating free radicals that oxidize unsaturated fatty acids. Despite literature [55,56,57] indicating an increasing pH trend during raw meat refrigerated storage due to endogenous or microbial enzyme action, like protease and lipase, resulting in increased volatile base concentration throughout prolonged storage [58], low pH during storage can be due to medium acidification caused by lactic bacteria growth, which generally occurs when meat is preserved in vacuum packaging [59,60].

## 4. Prooxidant Species and Preservation Properties of Bioactive Compounds in Meats and Their Products

Free radicals are atoms or molecules characterized by an uneven number of electrons, with one or more unpaired electrons in their outer shell, which renders them unstable and highly reactive. They form by donating an unpaired electron (oxidation) or receiving an electron from another molecule (reduction). The radicals achieve stability after the reaction, but the attacked molecule becomes a free radical, creating a chain reaction [43]. There are various types of free radicals, with oxygen-derived (O^2^) and nitric oxide (NO•) being the most significant in biological systems. Reactive species can be classified as radicals, such as superoxide radical anion (O_2_•^−^), hydroperoxyl radical (HOO•), and hydroxyl radical (HO•), or as non-radicals, including hydrogen peroxide (H_2_O_2_), singlet oxygen (^1^O_2_), and peroxynitrite anion (ONOO^−^). The generation of these reactive species is influenced by factors like unsaturated fatty acids, heme pigments, transition metal ions, and oxidizing enzymes, and they can also be produced in foods through irradiation and photo-oxidation [42,43,50].

ROSs are considered the most relevant as they can mediate cellular damage at increased concentrations. The superoxide anion (O_2_^−^), the hydroperoxyl radical (HO_2_), the hydroxyl radical (•OH) originating from H_2_O_2_, singlet oxygen (^1^O_2_), and the alkoxy radical (RO•) are examples of reactive species derived from molecular O_2_ metabolism [43]. When reactive species are produced in a concentration much higher than antioxidant systems can eliminate adequately, oxidative stress occurs due to system imbalance. Thus, biological macromolecules like lipids, proteins, and nucleic acids can be oxidatively damaged, causing numerous organic alterations and compromising cell viability. This process can occur in living biological systems or fatty-rich foods, i.e., animal-origin products [43].

An antioxidant is a substance that can prevent the oxidation of other molecules. In the context of food, antioxidants are natural or synthetic compounds that, in small amounts, can prevent, slow down, or delay the degradation of oxidizable substances, such as lipids and proteins, due to the action of atmospheric O_2_, thereby regulating the oxidation process [43,61]. In meat products, the increased surface area from greater fragmentation and the natural presence of microorganisms necessitate the use of methods such as refrigeration, freezing, modified atmospheres, and commonly synthetic preservatives or antioxidants by the food industry to manage oxidation and extend shelf life [48]. The definition of an antioxidant is used for any compound capable of delaying or preventing substrate oxidation. Antioxidants mitigate oxidative stress by safeguarding vital biomolecules from oxidative harm. Their actions include reducing molecular oxygen levels, eliminating pro-oxidant metal ions, neutralizing highly reactive species such as hydrogen peroxide and the superoxide anion radical, and deactivating singlet oxygen (^1^O_2_) [62].

Antioxidants can be categorized based on their mechanism of chain oxidation or their source. Regarding their role in the chain oxidation reaction, they can be classified as primary, being chain-breaking inhibitors, or secondary, being preventive [43]. As primary substances, antioxidants either transfer hydrogen atoms or donate electrons to neutralize or eliminate free radicals generated during the initiation or propagation phases, thereby deactivating the radicals and terminating the oxidation chain by forming stable compounds.

As secondary compounds, antioxidants function by mechanisms that reduce the rate of oxidation, such as binding metal ions (like iron and copper) that accelerate oxidation, substituting hydrogen with primary antioxidants, scavenging oxygen, deactivating singlet oxygen (^1^O_2_) through enzymes like glucose oxidase, superoxide dismutase, and catalase, absorbing UV radiation, and breaking down hydroperoxides [43,63].

**Figure 2:**
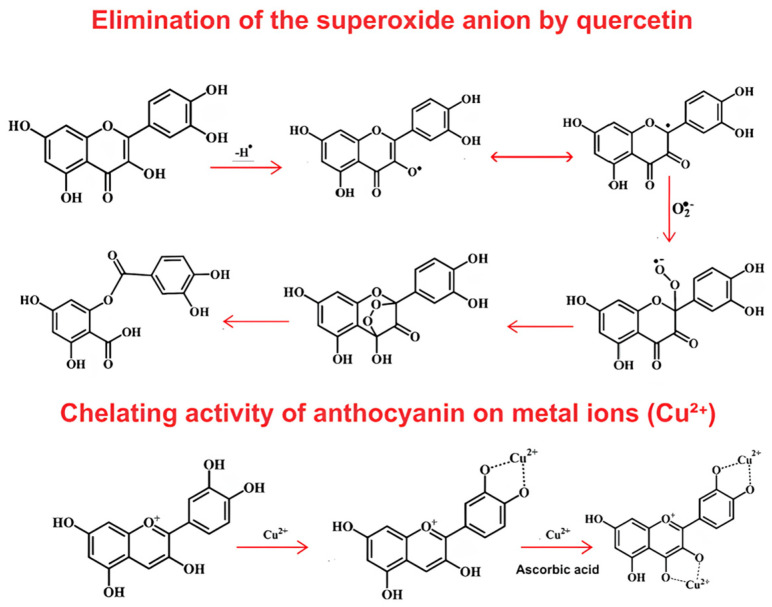
Antioxidant mechanisms of quercetin and anthocyanin flavonoids. Adapted from Nimse and Pal [65].

Among the natural antioxidants group, phenolic compounds have a significant representation. These secondary plant metabolites have benzene ring structures, with the main classes being phenolic acids and coumarins (including hydroxybenzoic acids, hydroxyl cinnamic acids, coumarins, and stilbenes), flavonoids (including flavonols, flavones, isoflavones, proanthocyanidins, and anthocyanins), and lignins. These compounds are unstable in the presence of heat and light and primarily have low bioavailability due to low solubility [64]. The antioxidant mechanism exercised by these compounds is exemplified in Figure 2.

Due to their phenolic hydroxyl groups, these compounds prevent or slow down the oxidation of lipids and other molecules by quickly donating a hydrogen atom or an electron to free radicals in a termination reaction, thus inhibiting the formation of new free radicals, such as superoxide and peroxyl ROS. They can also participate in scavenging lipid-derived carbonyls generated when lipids are oxidized in the presence of other food components, such as proteins and monosaccharides [63,66]. Another property contributing to the antioxidant capacity of phenolic compounds is the high tendency to chelate transition pro-oxidant metal ions responsible for initiating lipid degradation and scavenging reactive oxygen species [63].

**Figure 3:**
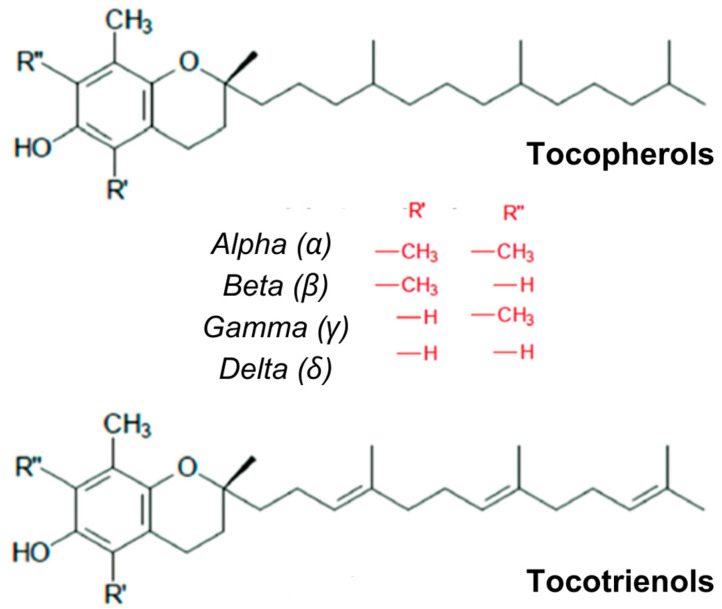
Tocopherols and tocotrienols classification. Adapted from Mcclements and Öztürk [67].

An important category of recognized natural antioxidants is tocols (tocopherols and tocotrienols). These compounds are defined as monophenolic compounds, constituting a series of chromanols (or methyl tocols). They occur in plant tissues such as green leaves and fatty foods, like vegetable oils, seeds, nuts, and egg yolk, being liposoluble antioxidants [48,61]. They comprise eight isoforms, or isomers, of chromanol, with four tocopherols (α-, β-, γ-, and δ-tocopherols) and four tocotrienols (α-, β-, γ-, and δ-tocotrienols), as shown in Figure 3.

Tocopherols include a 6-chromanol group and an apolar phytyl chain, bearing the prefix α-, β-, γ- or δ-, depending on the number and position of methyl groups attached to the chroman rings. The chroman group confers antioxidant activity to tocopherols, but the phytyl tail has no influence. All contain a chromanol ring with a hydroxyl (-OH) group capable of donating a hydrogen atom to neutralize free radicals, along with a hydrophobic side chain that facilitates their penetration into biological membranes [61]. Tocotrienols have a trienoid conjugated double bond system on the phytyl chains, while tocopherols do not. Methyl substitution affects vitamin E bioactivity, exercised by tocopherols, as well as their in vitro antioxidant activity [48,61].

Generally, it is assumed that the resulting tocopherol radical reacts with ascorbic acid (Vitamin C) at the lipid/aqueous interface, regenerating the tocopherol molecule. Meat-based foods containing relatively high concentrations of α-tocopherol demonstrate greater lipid and oxymyoglobin stability [48]. The hydrogen-donating power of tocopherols in fats, oils, and lipoproteins follows the order δ > β ≈ γ > α-tocopherol [61].

Due to their lipophilic character, tocopherols are located on membranes or with storage lipids, where they are immediately available to interact with lipid hydroperoxides. They react rapidly non-enzymatically to eliminate lipid peroxyl radicals, i.e., chain-carrying species propagating lipid peroxidation. In in vitro model systems, all tocopherols (α > γ > β > δ) and tocotrienols are good antioxidants [48].

α-tocopherol is a hydrophobic antioxidant, and this characteristic creates difficulties for experimental studies. This rekindles the use of α-tocopherol analogs that enable the capacity to work in homogeneous and aqueous solutions, besides having significant antioxidant activity. Of these, the trolox, where the α-tocopherol polyisoprenoid tail has been replaced by a carboxyl group, is reasonably water-soluble. In this molecule, the carboxyl group provides water solubility, while the antioxidant effect is provided by the chromanol group [61].

Regarding antimicrobial properties, the presence of medium-chain fatty acids (MCFAs) in vegetable oils is associated with microorganism inactivation. These saturated and unbranched monocarboxylic acids are especially present in coco and palm oils [68]. The MCFA group includes lauric acid (C12:0, dodecanoic acid), capric acid (C10:0, decanoic acid), caprylic acid (C8:0, octanoic acid), and caproic acid (C6:0, hexanoic acid). MCFAs have long been used in fodder preservation, especially in silage [68]. Fatty acids can cross bacterial cell membranes and thus cause conformational changes in the structure of the plasma membrane, affecting membrane permeability and disrupting electron transport, important for microbial cell survival [68]. Compounds jacalin and artocarpin, which are lectins present in jackfruit seed flour composition, demonstrated antimicrobial activity [69]. Furthermore, inhibition activity against pathogenic and spoilage microorganisms, like *Pseudomonas fluorescens*, *Salmonella enteritidis*, *Salmonella typhimurium*, and *S. aureus*, has already been reported for jackfruit co-products [70].

## 5. Synthetic Antioxidants in Meats and Their Products and Associated Complications

Data on using methods or food additives to prolong the shelf life of meats and enhance flavor registers since prehistoric humans, employing techniques like smoking, submersion in seawater, or centuries later with spice and sugar usage in fruit preservation [71]. Food additives are defined by the FDA as any substance used in the manufacture, processing, treatment, packaging, transportation, or storage of food, applied to improve safety, freshness, nutritional value, taste, texture, and appearance [71]. In Brazil, Resolution No. 778 defines a food additive as any substance intentionally added to food, not for nutritional purposes, but to alter its physical, chemical, biological, or sensory properties during stages such as handling, transportation, storage, packaging, treatment, preparation, processing, or production [72].

Although food additive use has become quite prominent in recent years due to increased processed food production, the consumer market has become more demanding regarding safe food additive use, seeking alternatives free from synthetic chemicals with biodegradable profiles [7,9]. This pressures food systems to become more innovative and sustainable in producing and processing nutritious and safe foods, as these characteristics are perceived by consumers as synonymous with health and determining factors for food acceptance [7].

Among the synthetic antioxidants, EDTA, TBHQ, PG, BHA, BHT, and sodium erythorbate are the most used in food. EDTA, in particular, serves as a chelating agent, binding to reducing metals and supporting the oxidation inhibition process [6,73]. BHT and BHA are antioxidants that delay rancidity in fats, sausages, and dried meats, while PG is commonly applied to prevent rancidity in processed fat products or pork sausages, being used in combination with antioxidants like BHA and BHT [71].

Different countries have their regulatory food systems and legislation, including allowed food additives and their maximum limits [43]. Table 1 presents the maximum levels of main synthetic antioxidants permitted for use in meat and meat products in the U.S., according to CFR no. 21/2023 and no. 9/2024 [24,74]; in Brazil, according to the Normative Instruction No. 211 [75], and in the European Union through Regulations no. 1333/2008 and no. 231/2012 [76,77].

Data obtained from online food sales platforms, physical retail stores, and the websites of prominent meat manufacturers were analyzed to investigate the types of preservatives used in beef meat products from major commercial brands. The analysis highlighted sodium erythorbate (INS 316) as the predominant preservative with antioxidant functionality in these products. Among the 24 items containing this additive, the distribution was as follows: beef meatballs (5), beef burgers (5), fiambre (4), beef kibe (3), and stuffed beef-based products (2). Notably, only one beef burger combined INS 316 with rosemary extract, while four products—two beef burgers and two beef meatballs—exclusively employed rosemary extract as a natural preservative. These results underscore the widespread use of synthetic additives in meat preservation while indicating a growing, though limited, shift toward natural alternatives with potential for broader adoption. The use of rosemary extract is the most suitable option due to its broad acceptance and compliance with regulations in various countries, offering greater assurance regarding international legislation.

Sodium erythorbate is the sodium salt of erythorbic acid, a highly refined, closely related to vitamin C chemical product synthesized from sugar and used as a color fixative in cured meat preparation [71]. It is a sodium ascorbate stereoisomer exhibiting antioxidant activity through singlet oxygen capture, hydrogen donation, and as a reducing agent [78]. As for safety, there is evidence suggesting that very high antioxidant levels, including sodium erythorbate, could potentially contribute to certain cancer developments, although this risk is considered minimal under typical dietary conditions. It is important to note that regulatory agencies have established acceptable daily intake levels to ensure consumer safety [78]. However, despite synthetic antioxidants being introduced to the food industry since 1940 with wide application, their use might be associated with toxicological effects [6,79]. The emergence of reports of toxicological adversities associated with many of these antioxidants has increased the demand for new sources of natural antioxidants, like flavonols, catechins, rosemary antioxidants, and herb extracts [43]. Also, the utilization of plant-extracted natural compounds as an alternative against multi-resistant bacteria. Food sanitization with chemical products like chlorine derivatives, iodophores, peracetic acid, and quaternary ammonium compounds is not always sufficient for prompt bacterial resistance promotion, driving the discovery of potential natural antimicrobial sources, especially in the food science field [7,8].

Due to the growing preference for safe preservatives in the market to control microorganisms, EOs are being increasingly used for food preservation and phytosanitary control, replacing traditional chemical preservatives. This shift comes as the overuse of chemical preservatives has become a contributing factor to the development of more resistant microbial strains [7,8].

Rosemary extract contains many polyphenols in its composition with great antioxidant power. Due to its safety, it’s considered a quantum satis additive, meaning it can be added to food in the necessary amount to achieve the intended technological task. Rosemary extract exemplifies how compounds from natural sources can be safely used to preserve food, even in synergy with other synthetic antioxidants, paving the way for seeking other natural food antioxidant sources [78].

The identification and utilization of natural antimicrobials and antioxidants present in EOs or other plant extracts, and their characterization of safety, specificity, and efficacy, but also applications of efficient delivery systems, represent fundamental objectives for the food industry [62]. Among the most commonly used natural antioxidants are tocopherols, phenolic acids, and plant extracts like rosemary and sage [48].

## 6. Essential and Edible Oils Applied to the Meat Industry and Its Products

EOs are secondary metabolites from different aromatic plants biosynthesized in various parts of the plant, like epidermal cells and glandular trichomes, and due to their high safety limits, they are recognized as generally recognized as safe (GRAS) substances by the FDA [10], considered harmless under prescribed usage conditions and exempt from the FDA’s testing and approval process [71]. The global EOs market was valued at USD 8.8 billion in 2022 and is projected to grow by 11.8%, reaching USD 15.3 billion by 2027 [80]. Additionally, their compounds, like phenolics and terpenes, are associated with antioxidant and antimicrobial effects [66].

Hydrocarbons are the main EO constituents, consisting of sesquiterpenes, terpenes, and different oxygenated compounds like phenols, aldehydes, lactones, esters, ketones, phenolic ethers, and alcohols. These compounds are generally chemically classified into two groups, (a) the main group containing terpenes and terpenoids and (b) the second group containing aromatic and aliphatic compounds [64]. Some of these constituents are widely known, such as cymene, geraniol, carvacrol, thymol, vanillin, gingerol, menthol, α-pinene, 1,8-cineole, eugenol, linalool, trans-anethole, and limonene [64]. These oils’ antimicrobial properties have also been reported against a wide range of microorganisms. Most exhibit greater inhibitory properties against Gram-positive than Gram-negative bacteria due to the lipopolysaccharide barrier in Gram-negative bacterial outer membranes [81]. Due to the hydrophobicity of its components, studies suggest EOs exert antimicrobial action by penetrating microbial membranes, causing ion leakage and cytoplasmic contents, thus inhibiting functional properties of the cell, along with causing interruption of molecular transport mechanisms, leading to inactivation, especially caused by terpenes [82].

Compounds like coumarins can reduce microorganism respiration. This demonstrates that not only a single compound would be responsible for antimicrobial activity, but also the combination of compounds can enhance bioactivity in synergistic action [48]. Several food preservatives containing EOs with both antimicrobial and antioxidant properties have been introduced to the market. One such product, “DMC base natural”, is a preservative made with a 50% blend of rosemary, sage, and citrus EOs and 50% glycerol, produced by DOMCA S.A in Spain [83]. Other examples include Guardian™ Rosemary Extract 201 and Guardian™ Green Tea Extract 20S, both of which are natural preservatives with antioxidant properties, derived from *Rosmarinus officinalis* and *Camellia sinensis* leaves, respectively. These products are part of the natural extract line by the European company Danisco S/A [84]. In Brazil, notable producers of EOs include Ferquima, Raros, and Dierberger, which manufacture oils for the food, pharmaceutical, cosmetic, and fragrance industries.

Antioxidant bioactive components found in EOs participate in safer and innovative formulations used as green or clean-label preservatives in food industries. Approximately 300 types of EOs from families such as *Cyperaceae*, *Piperaceae*, *Lauraceae*, *Myrtaceae*, *Lamiaceae*, *Apiaceae*, *Asteraceae*, and *Zingiberaceae* are commercially utilized across various industries [10]. *Rosmarinus officinalis* EO has antioxidant, antimicrobial, and anti-inflammatory properties, mainly due to the presence of monoterpenes such as 1,8-cineol, camphor, and α-pinene [85]. Other studies involving the application of EOs in meats and their products with preservatives (Table 2), highlighting these compounds’ antioxidant and antimicrobial potential.

**Figure 4:**
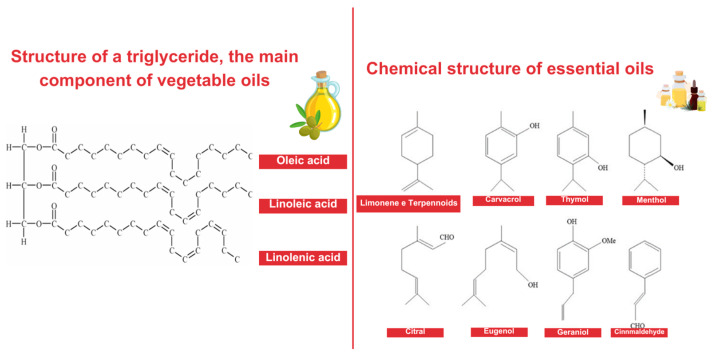
Differences between the chemical structures of vegetable oils and essential oils. Adapted from Dupain et al. [95] and Kashyap et al. [96].

The food industry seeks to explore essential oils’ preservative properties (antioxidant and antimicrobial) applied in meats and their products, being directly used in formulations or as an edible coating in different concentrations. The antimicrobial outcome is well explored, as well as the lipid and pigment (color) antioxidant properties. The edible oils (EdOs), unlike EOs, are composed mainly of fatty acid glycerides (Figure 4), containing small amounts of other lipids, such as phospholipids, unsaponifiable matter, or free fatty acids [90,91,92]. Regarding origin, they are classified as either animal, commonly extracted from fish produced in countries such as Peru, Vietnam, Chile, and the USA, or vegetable, extracted from plant sources, usually seeds or fruits, widely produced in countries like Indonesia, China, Malaysia, and Brazil [93,94].

EdOs play a crucial role in the human diet, as besides providing calories, they are sources of unsaturated fats, including essential fatty acids like linoleic, linolenic, and arachidonic acids, and also liposoluble vitamins (A, D, E, and K), offering various health benefits [91]. Unsaturated fatty acids in these oils have anti-inflammatory properties and help maintain cell membrane integrity. Liposoluble vitamins have vital roles in various biological processes, such as cellular growth regulation, bone health, vision, and blood clotting. For instance, vitamin E exerts an antioxidant effect, helping protect cells from oxidative stress, while vitamin K is essential for appropriate blood coagulation [97].

While animal oils are generally used in aquaculture and nutraceuticals, vegetable oils are used for food cooking, representing around 90% of worldwide oil production for human consumption. In industry, applications range from product formulation, like cakes, cookies, bread, margarines, and dairy products, to frying uses [91,93,94]. The world’s most-produced vegetable oils are palm oil (80.3 million tons), soybean (65.39 million tons), canola (34.02 million tons), and sunflower oil (21.74 million tons) [98]. Total vegetable oil production should exceed 228 million tons in 2024/25, with the highlight for soybean, palm, and canola oil.

EdOs, especially vegetables, have been used in industry as partial animal fat substitutes in meat products (Table 3), offering products with nutritionally balanced fatty acid profiles and reduced cholesterol content. A common approach to creating a stable meat mass is adding pre-emulsified oils; these oils are stabilized with a protein membrane, and they can be easily incorporated into ground meat matrices, where the oil droplets are stabilized by protein–protein interactions and function as fillers to improve products’ viscoelastic properties [35]. However, these changes meet limits in compromising product texture or increasing the lipid oxidation process, as unsaturated fatty acids are more susceptible to oxidation.

Depending on the oil type used, either essential or edible, these compounds can offer benefits such as shelf-life increase, reduction in oxidation, microbial growth inhibition, or nutritional profile improvement, responding to the food industry’s demand for natural and sustainable solutions. The application efficacy, however, varies according to the meat matrix, oil type and concentration, and application form, as shown in Table 2 and Table 3. EOs present high efficacy against microorganisms and oxidative processes in beef and lamb meats (Table 2). Similarly, EdOss-like portulaca seed, sweet pepper, and rice bran oils support oxidative or microbial preservation in meats (Table 3).

The EO of cinnamon (*Cinnamomum verum*) [86] presented antimicrobial efficacy in beef burgers, reducing *Aspergillus flavus* count and aflatoxin levels after nine days of refrigerated storage. Similarly, dill (*Anethum graveolens*) EO [28] showed lipid and protein oxidation inhibition, in addition to antimicrobial activity against *Enterobacteriaceae*, mesophilic aerobic, and lactic acid bacteria, indicating its appropriateness for preserving beef burger quality for up to three days of refrigerated storage.

Summer savory (*Satureja hortensis* L.) EO, applied in lamb meat combined in an edible coating, also demonstrated prolonged antioxidant and antimicrobial effects, inhibiting both bacterial cell and yeast and fungi growth, besides lipid oxidation, for up to 12 days [87]. This extended protection is promising as summer savory offers product shelf-life extension and reduced synthetic additives usage. Similarly, fennel (*Foeniculum vulgare*) EO applied in beef burgers promoted lipid and protein oxidation reduction and antimicrobial activity [29]. However, thyme (*Thymus vulgaris*) EO, when applied in beef sausages, showed variable effectiveness. In fermented sausages [89], thyme EO did not significantly inhibit microbial growth, suggesting its antimicrobial activity may be limited in low-acid environments or specific meat matrices, while inhibition of *S. aureus* and mesophilic aerobic bacteria growth was observed in raw sausages [88]. Rosemary (*Rosmarinus officinalis*) EO, applied in fermented sausages, presented higher efficacy in delaying lipid oxidation and better sensory acceptance [89] compared to thyme EO effects.

In terms of concentration, no optimal concentration has been determined to achieve prolonged effects, with varying results depending on the lipid complexity of the meat matrix. The use of low concentrations of EOs, between 0.15 and 0.31 μL/g, like dill and cinnamon [28,86] (Table 2), provided short-term antimicrobial effects for refrigerated products like burgers. However, thyme EO required a relatively high concentration (950 mg/g) to inhibit microorganisms in raw sausages, while other products like fermented sausages showed no significant microbial response, even at high concentrations [88] (Table 2). Some EOs, although effective in controlling oxidation and microbial activity, negatively impact product flavor and aroma, such as thyme oil in fermented sausages [89].

Some studies have also investigated the preservative properties of EdOs, such as portulaca seed oil [99], which promoted antimicrobial activity and lipid oxidation inhibition in beef burgers at the end of refrigerated storage; sweet pepper seed oil (*Capsicum annuum* L.) [101], which reduced lipid oxidation in beef burgers during ten days of refrigerated storage without significantly altering the sensory profile; and black rice bran oil [103] by inhibiting lipid oxidation and mesophilic bacteria count at the end of ground beef refrigerated storage. Other oils, such as walnut and safflower oils, applied in cooked beef burgers [102], improved the unsaturated fatty acid profile, with safflower oil increasing vitamin E content and reducing product hardness. These benefits are significant for developing healthier meat products that preserve texture and sensory quality. However, linseed, chia, and tiger nut oils, when used to replace fats in deer meat burgers [22], proved effective in reducing saturated fatty acids and increasing omega-3 for formulations containing linseed. However, all treatments with these oils showed an increase in lipid oxidation over storage, indicating the need for formulation improvements or the use of encapsulation techniques to reduce this long-term oxidative effect.

The essential and edible oils’ sensory impacts varied and were influenced by the concentrations and types of oil used (Table 2 and Table 3). Rosemary EO, for instance, was well accepted in fermented beef sausages [89] due to its natural aroma and antioxidant benefits. In contrast, formulations with chia oil, while increasing unsaturated fat levels, suffered a reduction in sensory acceptance [22]. Therefore, adjustments in formulations and encapsulation techniques are needed to mask strong flavors. Choosing between direct application or edible coatings can influence the different efficacy results, as can meat type and storage conditions. Specific combinations and controlled release technology can help maximize benefits. Techniques like nanoencapsulation of these oils aim to minimize usage limitations, allowing controlled release and prolonged antioxidant protection without significantly compromising flavor.

Essential and edible oils are among the group of nutrients, hydrophobic foods, or poorly soluble compounds. Hence a series of factors limit these ingredients’ application in foods, including low stability due to oxygen, light, and temperature sensitivity; low solubility; and low bioavailability [64]. Moreover, EOs, fish oil, crude palm oil, and other oils possess marked flavor properties limiting their direct formulation addition due to their influence on sensory food quality [64].

Recently, various encapsulation technologies have been recommended to enhance these oils’ bioefficacy [10]. Therefore, nanoencapsulation is an alternative to control these limitations, increasing solubility and bioavailability. This technology acts by protecting unstable components, like bioactive compounds, from unfavorable conditions during processing, storage, and transport to improve quality and stability, as well as improving bioactivity, allowing controlled compound release, and masking undesirable tastes in foods [64].

## 7. Nanoencapsulation of Essential and Edible Oils

**Figure 5:**
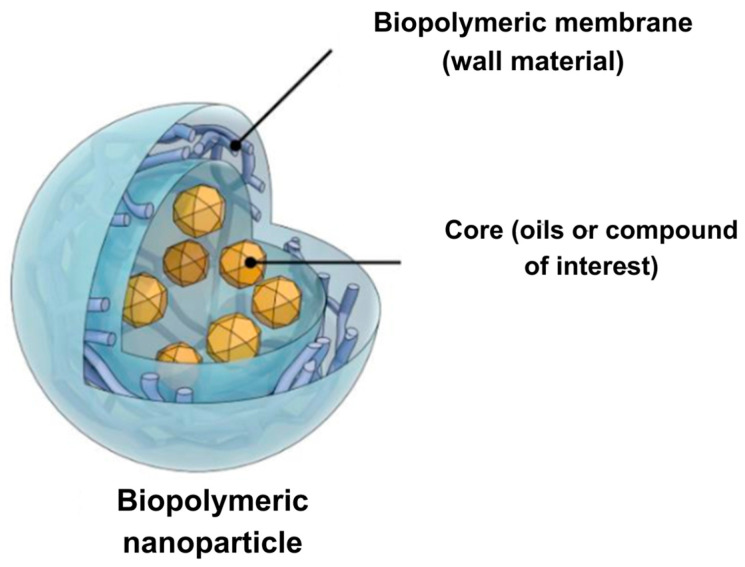
Structure of a biopolymeric nanoparticle. Source: Adapted from Bayraktar et al. [63].

Nanoencapsulation is a method in which one or more ingredients are immobilized in some form of matrix or wall. This technology allows entrapping interest material (e.g., oils) within a protective polymeric covering at the nano-range, i.e., 10–1000 nm. The encapsulated material is known as core, internal phase, or active agent, while encapsulation substances are recognized as wall material, carrier agent, external phase, capsule, carrier material, or matrices [10,63]. Such nanoparticles can be lipophilic or hydrophobic depending on the incorporated materials and the method used to obtain them [108]. Nanoparticles with wall material (Figure 5) are an intelligent delivery system that increases the physical stability and bioavailability of bioactive compounds (vitamins, antioxidants, proteins, and lipids, as well as carbohydrates) [62,109], thus gaining interest in pharmaceutical and food domains [62,109]. In this system, protection from aggressive environmental factors and controlled release of core compounds is achieved [62]. Reducing particle size improves delivery properties, solubility, and bioavailability of the nutraceutical, even compared to microencapsulation, since delivery properties can be enhanced due to greater surface area per unit volume achieved in a nanosystem [63].

**Figure 6:**
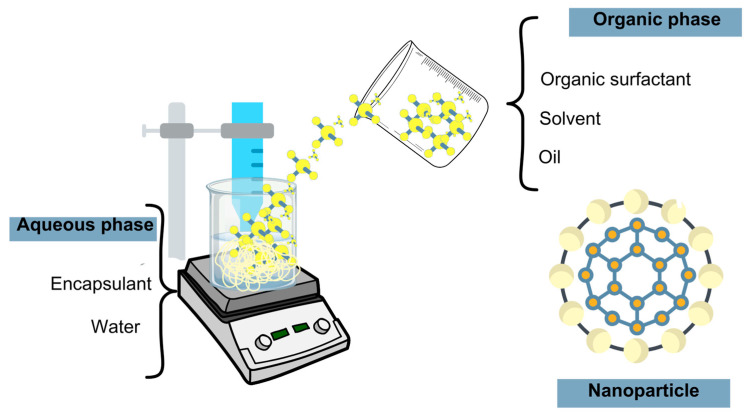
Obtaining polymeric nanoparticle by homogenization. Adapted from Kedia and Dubey [117].

This technology added a new dimension to food industries by increasing oils’ bioefficacy, which, in nanostructures, increases solubility, targeted delivery capacity, and active component long-action relief, thus increasing long-term properties and reducing the negative food flavor impact [10,82,110]. Regarding nanotechnology utilization and studies with edible and EOs, nanoparticles were developed containing nanoencapsulated fish oil [111,112], palm olein [113], crude palm oil [114] and its stearin and olein fractions [115], chia seed oil [116], and EOs from different plants [9,112] for various purposes. Different technologies can be employed to obtain nanoparticles encapsulated by biodegradable polymers, and derived interactions are several, like electrostatic attraction, hydrogen bonds, hydrophobic interactions, etc. [62]. Amongst this diversity of methods, techniques like homogenization, nanoprecipitation, and ionic gelation are widely used for oil encapsulation [10]. In homogenization (Figure 6), the organic phase (usually composed of organic surfactant, solvent, and oil) is slowly dripped into an aqueous phase containing an encapsulant solution in water, and then the solvent undergoes evaporation [92,116].

The ideal characteristics associated with successful nanoparticle production include smaller size, higher surface charge, and lower polydispersity index (PDI) [9]. The average diameter typically increases due to the swelling of polymeric nanoparticles as more oil is added to the formulation [109,118]. The PDI is another crucial parameter when evaluating nanoparticle production; values below 0.2 are considered ideal, while values above 0.5 indicate a very broad size distribution [7]. Encapsulation efficiency is affected by several factors, such as the type of wall material, the core material properties (concentration and volatility), and the characteristics of the encapsulate (total solids, viscosity, and droplet size) [119].

The zeta potential (ZP) analysis provides valuable insights into the stability of aqueous nanosuspensions against undesirable phenomena such as precipitation or flocculation [7]. The zeta potential is commonly correlated with the stability of dispersed systems, classified as ±0–10 mV (very unstable), ±10–20 mV (relatively stable), ±20–30 mV (moderately stable), and greater than ±30 mV (highly stable) [120]. According to the electrostatic DLVO theory (Derjaguin, Landau, Verwey, and Overbeek), there is a balance between Van der Waals attractive forces and electrical repulsion due to net surface charge. A higher surface charge on nanoparticles prevents aggregation due to strong repelling forces between particles. Absolute ZP values above 30 mV confer good stability, while values around 20 mV offer only short-term stability, and values around 5 mV indicate rapid aggregation [121].

Despite these ideal parameters, ZP values below 30 mV have been reported in the literature for stable nanoparticles, owing to the absence of aggregation. In addition to the zeta potential effects, the steric stabilization provided by hydrophilic surfactants, such as Tween 20 or Tween 80, when enveloping the encapsulating polymer or biopolymer, plays a crucial role in inhibiting initial nanoparticle coalescence, contributing to system stability [7,121,122].

Solvents used in nanoparticle preparation must be water-miscible, making acetone a common choice. However, as it is considered toxic, cereal alcohol is emerging as a safe (Generally Recognized as Safe—GRAS) alternative for food use. Furthermore, the growing demand for natural, healthy, and sustainable foods has fueled global interest in minimizing environmental impacts [123].

The use of nanoparticles is associated with a range of parameters, including security, environmental impact, and regulation, with variations in regulatory aspects across countries regarding foods developed with nanotechnology. In the European Union, nanomaterials have been regulated by the REACH regulation (Registration, Evaluation, Authorization, and Restriction of Chemicals) since 2020, whereas in the United States, regulation is sectoral, with the Food and Drug Administration (FDA) overseeing nanotechnology use in foods [124]. In Brazil, the Collegiate Board Resolution (RDC) No. 839 of 2023 introduced updates related to safety proof and authorization for the use of new foods and ingredients, including those developed with nanomaterials [125]. This RDC mandates that foods made with nanomaterials must have safety proof, encompassing detailed characterization of elemental chemical composition, morphology, particle structure, particle size distribution, surface properties, and specific toxicological studies, such as research on cytotoxicity/cell viability, oxidative stress induction, toxicokinetics, genotoxicity, and subchronic, among others [125].

The impact of nanoparticles on human health (nanotoxicity) is influenced by their chemical composition, size, mass, and surface properties. Pisoschi et al. [62] indicate that nanoparticles made from biopolymer materials exhibit better cell and tissue compatibility, gaining interest among researchers due to their therapeutic properties and enhanced stability in biological fluids during storage [126].

A variety of biopolymers have been used as encapsulating materials for nanoparticles, including natural macromolecules (chitosan, alginate, gelatin, cellulose, etc.), synthetic biopolymers like poly(lactic acid), poly(lactic-co-glycolic acid), polyhydroxybutyrate, and derivatives of these materials. Biopolymeric nanoparticles can also be synthesized from food product biopolymers [62]. Research dedicated to synthesizing biodegradable biopolymers began in the early 1930s with polylactic acid, but challenges such as high costs and hydrolysis susceptibility were encountered [62]. Other biopolymers used are food-based, like proteins or polysaccharides, which produce various nanostructures encapsulating functional ingredients [62].

### Nanoparticles of Essential and Edible Oils Applied in the Meat Industry

The main applications and purposes of EO nanoparticles extend from antimicrobial and antioxidant activity to preserving sensory properties and bioactive compounds [9,112]. As shown in Table 4, these nanoparticles have diverse applications in the meat industry, including studies involving beef hamburgers [86,127,128], ground beef [129,130], and beef sausages [131].

As observed in Table 4, applications targeting shelf-life extension are not limited to the direct addition of nanoparticles to formulations but also involve the use of edible coatings, either through immersion or film addition. Lipid oxidation inhibition is the main antioxidant effect explored for nanoparticles of EOs applied to meats and their products. Investigating the prevention of protein and pigment oxidation is also a focal point of interest.

Studies in Table 4 indicated that EO nanoparticles are generally more effective than free EOs in controlling/inhibiting oxidative processes and providing antimicrobial effects without imposing negative sensory properties on products. Studies demonstrate greater inhibition of microorganism growth, coupled with favorable sensory evaluations [86]. However, increased lipid oxidation may occur in products containing high concentrations of unsaturated compounds [132].

EO nanoparticles applied to meat products showed promising results in controlling microorganisms and oxidative preservation, achieving effective outcomes compared to free oil forms, with varied impacts on sensory acceptance. Generally, there is a common trend among studies focused on EOs like cinnamon, lemongrass, thyme, and garlic that aim for both antimicrobial and antioxidant effects (Table 4). However, variations in formulations, concentrations, meat matrices, and sensory impact seem to affect different results, highlighting both the effectiveness and challenges of these technologies. These oils are applied through edible films or nanoemulsions, forming barriers that restrict oxygen contact with lipids, reducing lipid oxidation, and preserving sensory quality and meat color stability during storage.

Cinnamon EO [86], applied to beef hamburgers at a 0.156 μL/g concentration, effectively inhibited *Aspergillus flavus* growth, reduced aflatoxin levels, and showed good sensory acceptance. Similarly, lemongrass EO in nanoemulsion (1.5 mL/g) applied to beef hamburgers [127] was also effective, inhibiting yeast and mold growth, *E. coli*, and *S. aureus*, and delaying lipid oxidation over 4 months of freezing, maintaining good sensory acceptance. The trend continues with thyme EO [128], applied to beef hamburgers at a 10 mg/g concentration in a casein-maltodextrin matrix, inhibiting coliform *E. coli* growth over 14 days of refrigerated storage. In ground beef, *Cinnamodendron dinisii* Schwanke EO effectively inhibited lipid and myoglobin oxidation, preserving the red color for 12 days [130]. These examples illustrate the consistent efficacy of different EOs in meat products, allowing for microbial proliferation inhibition and slowing oxidative processes.

While antimicrobial and antioxidant properties are common, there are differences regarding sensory impacts and application forms. In beef sausages, garlic EO encapsulated in a 2% chitosan film inhibited aerobic and psychrophilic bacteria growth over 50 days of refrigerated storage. However, encapsulated garlic did not provide additional sensory benefits over free EO, indicating limitations in flavor and texture improvements [131].

Zataria multiflora EO [132], applied to ground beef at 1% concentration, offered a notable advantage: besides inhibiting lipid and protein oxidation, it did not negatively alter flavor, odor, or meat color. This lack of undesirable sensory impact contrasts with garlic oil, highlighting that EO type and encapsulation method can influence product acceptance.

In terms of the use of EdOs nanoparticles in developing new meat products, multiple studies have employed different meat types treated with nanotechnology to enhance nutritional value or technological improvements, including beef hamburgers [86,127,133,134,135] and lamb loins [136,137]. Thus, the use of EdOs nanoparticles in meat products has shown satisfying outcomes in increasing durability and quality, given their minimal influences on sensory parameters. The application of these nanoparticles for nutritional profile enhancement and even increased oxidative stability of meat products is a general trend in studies (Table 5), which explore various matrix and encapsulation combinations, aiming for both oxidative preservation and improved nutritional value. These oils are frequently used as fat substitutes, potentially benefiting product texture, moisture retention, and lipid profile. However, the studies report variations in antioxidant efficacy, sensory acceptance, and nutritional impact, reflecting differences in formulations and oil types used.

The inclusion of EdOs nanoparticles in meat products can occur directly in formulation or as an edible coating (Table 5). Interest in their application varies between nutritional improvement (fat substitutes) or preservative effects of active compounds in oils. A challenge identified by these studies is the need to prevent lipid oxidation, more pronounced when replacing animal fat with EdOs nanoparticles due to the high content of unsaturated fatty acids added to the formulation. Fish oil, for example, added to beef hamburgers in nanoparticle form, partially replacing fat, resulted in reduced caloric content, higher water retention, and inhibited lipid and protein oxidation during refrigerated storage [133]. These benefits were accompanied by improved texture and color with good overall sensory acceptance. Similarly, safflower oil was added to beef hamburgers in the form of an oil-based nanoparticle hydrogel blended with konjac flour and sodium alginate, replacing 100% of the fat [134]. Despite promoting lipid oxidation, this formulation increased polyunsaturated fatty acid percentage and reduced the product’s atherogenic index, maintaining good sensory acceptance and enhancing hamburger texture.

Acai oil was used in the form of a lyophilized hydrogel based on oil nanoparticles and a konjac flour/sodium alginate blend, partially replacing fat in beef hamburgers, improving unsaturated fatty acid content, and reducing saturated fats [135]. The authors observed an improvement in yield and texture parameters and good sensory acceptance without significant differences from the control, even though it also promoted lipid oxidation, like fish oil.

Some of these studies investigated the effect of using isolated EdOs nanoparticles combined with EO, demonstrating that combining nanotechnology and EdOs is viable not only for nutritional value improvement but also for targeted antioxidant and antimicrobial action. When combined with EOs, this protective and shelf-life-extending potential is more pronounced. These results were observed in studies conducted on lamb loin by researchers Hasani–Javanmardi; Fallah and Abbasvali [136], who used sunflower oil and cumin EO, and a study by Jafarinia; Fallah, and Dehkordi [137], which combined olive oil and ajowan (*Carum copticum*) EO effects (Table 5). Another frequently evaluated feature in Table 5 articles is the impact of nanoparticle use on product texture and flavor, particularly when using oils with strong sensory properties like fish oil [133]. In the study by Hanula et al. [135], beef hamburger samples containing açaí oil nanoparticles showed good color stability but increased lipid oxidation, indicating that fat replacement can negatively impact oxidative stability despite nutritional benefits.

## 8. Conclusions

Replacing synthetic with natural antioxidants is a promising approach to control lipid oxidation and microbial contamination in meat and its products, particularly hamburgers. Moreover, consumers have pressured food systems to offer more natural products. Among natural antioxidants, essential oils are explored as alternatives to synthetic preservatives, raising toxicological concerns. Additionally, EdOs, beyond their nutritional improvement potential, are already employed for preservation purposes. These oils, rich in bioactive compounds, decrease microbial deterioration and lipid oxidation and provide a clean-label option aligned with food safety and sustainability requirements. However, directly using oils presents challenges such as low solubility and sensory alteration, which can be mitigated through encapsulation technologies. Nanoencapsulation can benefit oil utilization in the meat industry and its products, broadening industrial application by preserving bioactive compounds and minimizing strong flavor effects. The combination of edible films and nanoparticles shows effective results in extending shelf life, microbial control, and maintaining sensory quality. However, further research is needed to refine application methods and meet sensory preferences, aiming for more sustainable and acceptable solutions for the food industry.

## Figures and Tables

**Table 1:** Maximum levels of the main synthetic antioxidants allowed in meats and their fresh processed products in different countries.

Synthetic Antioxidant (Maximum Levels—mg/kg)					Reference
Sodium Erythorbate (316)	BHA (320)	PG (310)	BHT (321)	TBQH (319)	
Quantum satis	1000	1000	Not reported	Not reported	[75]
500	100	100	100	200	[24,74]
500	200	200	200	200	[76,77]

BHT: butyl-hydroxytoluene, BHA: butyl-hydroxyanisole, PG: propyl-gallate, TBHQ: terc-butyl-hidroquinone; Quantum satis: minimum concentration of the food additive or adjuvant of sufficient technology to obtain the desired effect.

**Table 2:** Effects of essential oil application in meat and their products.

Meat or Meat Product	Essential Oil (EO) Source	Evaluated Properties	Application Form	Concentration of EO	Highlights	Reference
Raw beef burger	Cinnamon (*Cinnamomum verum*)	Antimicrobial activity	Free EO	0.31 μL/g	Reduction of *Aspergillus flavus* counts inoculated in meat at 9 days of cold storage and reduction of aflatoxin levels at 8 days of storage.	[86]
Raw beef burger	Dill (*Anethum graveolens*)	Antioxidant and antimicrobial activities	Free EO	0.15 µL/g	Inhibition of lipid and protein oxidation and antimicrobial potential against *Enterobacteriaceae*, mesophilic aerobic bacteria, and more strongly against lactic acid bacteria, in 3 days of cold storage.	[28]
Raw lamb burger	Savory (*Satureja hortensis* L.)	Antioxidant and antimicrobial activities	Edible Coating	1.5% savory EO solution with seed mucilage of *Lepidium sativum*	Growth inhibition of total cells, psychrophilic bacteria, *Escherichia coli*, *S.aureus*, fungi and yeasts for 12 days of cold storage. Inhibition of lipid oxidation and extension of shelf life of meat to 12 days of cold storage.	[87]
Raw beef burger	Fennel (*Foeniculum vulgare*)	Antioxidant and antimicrobial activities	Free EO	0.150 μL/g	Reduction of lipid and protein oxidation, growth of mesophilic aerobic bacteria, lactic acid bacteria and *Enterobacteriaceae* in 3 days of cold storage. Efficient inhibition of *Escherichia coli* growth and discrete inhibition of *Listeria monocytogenes*, both inoculated in 3 days of cold storage.	[29]
Raw beef sausage	Thyme (*Thymus vulgaris*, L.)	Antimicrobial activity	Free EO	950 mg/g	Inhibition against coagulase-positive *Staphylococcus* and aerobic bacteria. Absence of inhibition against thermotolerant coliforms in 15 days of cold storage.	[88]
Raw beef sausage (turk sausage or sucuk)	Thyme (*Thymus zygis*) and rosemary (*Rosmarinus officinalis*)	Antioxidant and antimicrobial activities	Edible Coating	1% thyme or rosemary EO solution with chitosan	Absence of inhibition on fungal growth and aerobic bacteria, lactic acid, Gram^+^, Catalase^+^ and *Enterobacteriaceae* cocci during 3 months of cold storage. Inhibition of lipid oxidation at the end of storage, with no statistical difference between EO sources. Both treatments showed acceptable sensory attributes at the end of storage, with emphasis on a higher odor score in sausages with the rosemary treatment.	[89]

**Table 3:** Effects of edible oil (EdO) applications on meats and meat products.

Meat or Meat Product	Edible Oil	Evaluated Properties	Application Form	Concentration of EdO	Highlights	Reference
Raw beef burger	Purslane seed oil (*Porlutaca oleracea*)	Antioxidant and antimicrobial activities	Edible Coating	3% inclusion with soy protein isolate	Inhibition of the growth of *S. aureus*, *Bacillus cereus* during 15 days of cold storage;. Inhibition of lipid oxidation at the end of cold storage.	[99]
Beef sausages	Refined sunflower oil	Improved nutritional value	Free EdO	100% fat substitution	Increased hardness, but no changes in physical and textural properties; Increased fat retention after cooking.	[100]
Raw beef burger	Sweet pepper seed oil (*Capsicum annuum* L.)	Antioxidant activity	Free EdO	1.6 g/g	Inhibition of lipid oxidation in 10 days of cold storage.	[101]
Cooked beef burger	Walnut oil Safflower oil value	Improved nutritional	Free EdO	100% fat substitution	Increase of unsaturated fatty acid content for samples containing both oil sources, but higher unsaturated content for safflower oil oven-cooked at the end of 60 days of frozen storage at −20 °C; Increase of vitamin E levels in samples containing walnut oil at the end of frozen storage at −20 °C; Increased oxidation inhibition, lower hardness, higher cooking yield, and improved sensory evaluation scores for samples containing safflower oil.	[102]
Goat meat burger	Olive oil (AO) and sunflower oil (OG)	Improved nutritional value	Hydrogel emulsion with 37.3% of EdO	4% fat substitution	Reduction of saturated fat content and improvement of fatty acid profile for both formulations, with emphasis on samples containing olive oil. Highest b* (yellow-blue intensity) color parameter value for samples containing olive oil. Inhibition of lipid oxidation during 3 days of cold storage for cooked hamburgers containing hydrogel emulsion, with no statistical difference between both oil sources.	[20]
Ground beef	Black rice bran oil	Antioxidant and antimicrobial activities	Free EdO	0.4 mL/g	Inhibition of lipid oxidation at the end of 6 days of cold storage; Inhibition of mesophilic bacteria count at the end of storage.	[103]
Mutton sausage	Flaxseed oil	Improved nutritional value	Preemulsion with flaxseed oil plus soy protein isolate (1:2)	40% fat substitution	Higher a* (red-green intensity) color parameter value for samples containing flaxseed oil compared to control in 60 days of storage at −18 °C. Greater lipid oxidation compared to end-of-storage control.	[104]
Venison Burger	Tiger nut oil (*Cyperus esculenus*), chia oil and linseed oil	Improved nutritional value	Free EdO	100% fat substitution	Reduction in saturated fatty acid content in all treatments; Increased polyunsaturated and omega-3 fatty acids and reduced n-3/n-6 ratio for formulations with linseed oil; Increase in monounsaturated FA content for tiger nut oil formulations; Good overall acceptance for samples with the addition of tiger nut oil or linseed oil, but low for samples containing chia oil; Increased lipid oxidation in all treatments, but more pronounced in samples containing chia oil at the end of 18 days of cold storage; No significant differences in texture between treatments and the control sample.	[22]
Beef burger	Blend of chia and flaxseed oil	Improved nutritional value	Hydrogel emulsion with 25% of oil blend (12.5% each)	60% fat substitution	Reduction of 25% of total lipid content and more than 30% of saturated fatty acids; Increased omega-3 content; Increased lipid oxidation (raw and cooked samples); No change in parameters L* (luminosity), a*, b* in raw samples and an increase in b* for cooked samples; Maintenance of yield, moisture, and diameter, and increased hardness, chewability and lipid retention; Good overall sensory acceptance;	[21]
Unfermented beef (*Frankfurt* sausage)	Palm kernel oil	Improved nutritional value	Free EdO	100% fat substitution	Increase in caprylic, capric, lauric, and myristic acid content and a decrease in palmitic, palmitoleic, stearic, and oleic acid levels; Increased lipid oxidation after manufacturing and after 30 days of cold storage; Absence of impact on moisture, fat, protein, ash, and sensory properties; Reduction in texture quality; Good overall sensory acceptance.	[105]
Fermented beef sausage (Turk sausage or “sucuk”)	Palm kernel oil	Improved nutritional value	Free EdO	100% fat substitution	Reduction of total lipid and protein content; Increase in caprylic, capric, lauric, and myristic acid content and decrease in palmitoleic, stearic, oleic, and linoleic acid; Low impact on increased lipid oxidation after 30 days of cold storage; Increase in a* and b* values at the end of fermentation; Reduction in texture quality;	[106]
Ground beef and liver	Refined palm oil	Improved nutritional value	Free EdO	20% fat substitution	Maintenance of water holding capacity, yield, and texture similar to control; Good overall sensory acceptance, no difference with the controller.	[107]

**Table 4:** Effects of application of essential oil (EO) nanoparticles in meat and their products.

Meat or Meat Product	Essential Oil	Evaluated Properties	Application Form	Concentration EO	Highlights	Reference
Beef burger	Cinnamon (*Cinnamomum verum*) Antimicrobial activity	Antimicrobial activity	Addition of cinnamon EO nanoparticle with chitosan and pectin	0.156 μL/g	Greater inhibition in the growth of *Aspergillus flavus* in relation to control; and samples containing free cinnamon EO had no counts until the 9th day of cold storage. Greater reduction in aflatoxin levels (68.2%) than free EO (40.9%). Good sensory acceptance.	[86]
Beef burger	Lemongrass (*Cymbopogon citratus*)	Antioxidant and antimicrobial activities	EO Nanoemulsion addition	1.5 mL/g	No inhibitory effect for mesophilic aerobias over 4-month storage under freezing. Inhibition of the growth of fungi, *Escherichia coli*, and *S. aureus* at the end of storage. Increased inhibition of lipid oxidation at the end of storage. Increase in cooking properties at the end of storage. Good overall sensory acceptance, taste, aroma, texture.	[127]
Ground beef	*Cinnamodendron dinisii* Schwanke	Antioxidant activity	Edible Coating	Film at 3.69 μL/mL of EO and zein nanoparticle	Inhibition of lipid oxidation and myoglobin (maintenance of red color) over 12 days of cold storage.	[130]
Beef burger	Thyme (*Thymus vulgaris*)	Antimicrobial activity	Addition of thyme EO and casein-maltodextrin nanoparticles	10 mg/g	Inhibition of the growth of thermotolerant coliforms and *Escherichia coli* during 14 days of cold storage.	[128]
Beef sausages	Garlic	Antioxidant and antimicrobial activities	Edible Coating	2% EO nanoparticle film with chitosan	Inhibition of the growth of aerobic, psychrophilic, and lactic acid bacteria over 50 days of vacuum-refrigerated storage. Inhibition of lipid oxidation at the end of storage. Absence of better sensory properties than samples containing EOL in free form.	[131]
Ground beef	*Zataria multiflora* Antioxidant and antimicrobial activities	Antioxidant and antimicrobial activities	Edible Coating Film at 1% EO nanoemulsion	Film at 1% EO nanoemulsion	Inhibition of microbial growth. Inhibition of lipid and protein oxidation over 20 days of cold storage. Absence of negative changes in taste, odor, color, and overall acceptability. Absence of significant differences in sensory properties compared to samples containing the free oil.	[132]

**Table 5:** Effects of the application of edible oil nanoparticles in meats and their products.

Meat or Meat Product	Edible Oil (EdO)	Evaluated Properties	Application Form	Concentration	Highlights	Reference
Beef burger	Fish oil	Improved nutritional value	Nanoparticle with 10% fish oil	Fat Substitute *	Reduction of total calories; Inhibition of lipid and protein oxidation; Increased water holding capacity and cooking efficiency; Better parameters for texture and color; Highest score on texture, taste, smell, color, and overall acceptance.	[133]
Beef burger	Safflower oil	Improved nutritional value	Hydrogel based on safflower oil nanoparticles (45%) and a blend of konjac flour and sodium alginate, enriched with acai extract	100% fat substitute (95 mg/g)	Promotion of lipid oxidation during 8 days of cold storage; Improved chewability and hardness, No change in color; Increased percentage of polyunsaturated fatty acids; Reduction of atherogenic and thrombogenicity index; Increase in hamburger hypocholesterolemic/hypercholesterolemic ratio.	[134]
Beef burger	Acai oil	Improved nutritional value	Lyophilized hydrogel with acai oil nanoparticles (31%) and a blend of konjac flour and sodium alginate	50% fat substitute	Increase lipid oxidation during 7 days of cold storage relative to control; Greater stability of red colors (positive values in parameter a*) at the end of storage compared to the control; Increase in polyunsaturated fatty acid content and reduction of saturated fatty acids; Reduction of atherogenic and thrombogenicity index; Increase in hamburger hypocholesterolemic/hypercholesterolemic ratio; Improved yield and texture parameters; Good sensory acceptance, with no significant difference from control.	[135]
Lamb loin meat	Sunflower oil and Cumin essential oil	Antioxidant and antimicrobial activities	Edible coating	Sunflower oil nanoparticle solution and the addition of 1% cumin essential oil	Inhibition of the growth of mesophilic aerobic bacteria, psychotrophs, Enterobacteriaceae and lactic acid bacteria during 20 days of cold storage, for both treatments, with greater inhibition with the addition of both oils; Inhibition of the total volatile nitrogen content formed, lipid and protein oxidation, inhibition of metamyoglobin formation, and color deterioration at the end of storage for both treatments, with greater inhibition with the addition of both oils; Good sensory acceptance of up to 16 days of storage for sunflower oil samples and up to 20 days for samples with both oil sources.	[136]
Lamb loin meat	Olive oil and ajowan essential oil (*Carum copticum*)	Antioxidant and antimicrobial activities	Edible coating	Olive oil nanoparticle solution and the addition of 2% ajowan essential oil	Inhibition of the growth of mesophilic aerobic bacteria, psychotrophs, Enterobacteriaceae, and lactic acid bacteria during 16 days of cold storage for both treatments, with greater inhibition with the addition of both oils; Inhibition of the total volatile nitrogen content formed, lipid and protein oxidation, inhibition of metamyoglobin formation, and color deterioration at the end of storage for both treatments, with greater inhibition with the addition of both oils; Good sensory acceptance up to 12 days of storage for olive oil samples and up to 16 days for olive oil plus +2% ajowan essential oil samples.	[137]

* Not reported.

## Data Availability

No new data were created or analyzed in this study. Data sharing is not applicable to this article.
